# Dr. Blake on Delirium Tremens

**Published:** 1830-07-01

**Authors:** 


A PRACTICAL ESSAY ON DELIRIUM TREMENS, &c. By ANDREW BLAKE, M.D. &c. Octavo, pp. 68. 1830.

In the Edinburgh Medical and Surgical Journal for October, 1823, is a paper on the subject of this Essay by the same author. In the next year or two, Dr. Blake, while surgeon of the fifth regiment, saw many cases of the disease in the islands of St. Vincent and Dominica, all of which tended to confirm him in the opinions he had previously entertained as to its nature and treatment. The result of Dr. B.'s whole experience formed the subject of a thesis for graduation at Glasgow.

Our author is dissatisfied with the names which have hitherto been conferred on this peculiar malady—even with the one first used by himself—delirium ebriositatis. He now thinks the term ERETHISMUS EBRIOSITATIS the best designation, offering the following as the definition of the disease. "Indirect general debility, succeeded by a morbid increase of action in the brain and nervous system, which is attended with delirium, and terminates generally, either in sleep and subsequent health, or in death from collapse or effusion on the brain."

The complaint is very prevalent among British soldiers, as well as the lower of the white population between the tropics—no doubt on account of the facility of procuring ardent spirits there. The author, after some preliminary observations, proceeds to divide the disease into three distinct stages—succeeding each other as regularly as the stadia of an ague, "to which indeed it bears no small analogy, being to the brain and nerves what intermittent fever is to the arterial system." In ten cases, to which he alludes in a table, the stage of nervous excitement came on at different periods, within five or six days from the date of admission for other complaints. It succeeded to trivial affections, of various sorts, and the period in hospital might be considered, in general, as the date at which the patient began to abstain from intemperate habits. Dr. B. has had subsequent opportunities of seeing the disease come on during the prevalence of endemic remittent fever and ophthalmia, as well as different kinds of accidents. Patients taken into hospitals are, of course, deprived of spirituous potations, and the consequence is, that the nervous system of a person accustomed to such stimulation feels the loss of it, and gradually sinks into a state of extreme exhaustion, generally increased by the depletory measures employed for the other complaint. This state of exhaustion is the first stage of the malady, and is succeeded by a state of delirium too easily confounded with phrenitis, and too often fatal when so confounded.

The first distinct indications of this disease, according to our author, are, a peculiar slowness of the pulse, attended with coldness of the hands and feet, as also a cold moisture. These are preceded or accompanied by general debility, diminished temperature, and occasionally by cramp in the muscles of the lower extremities, giddiness, nausea, sometimes vomiting. The bowels are usually open, sometimes confined. There is nervous tremor of the hands and tongue, the latter being moist and but slightly furred. To these may be added dejection of spirits, frequent sighing, oppression of the præcordia, anxiety, short and interrupted slumbers.

As the second stage approaches, the countenance gradually assumes a wild aspect, the patient becomes restless, with an apparent anxiety to perform whatever you desire. The duration of the first stage will be in proportion to the nature and extent of the cause, and the state of the constitution and previous habits of the individual.

"When the second stage is established, a train of symptoms consonant with high nervous irritation gradually follows; mental alienation, in various degrees and forms, is developed, and with this an exertion of the nervous power to re-establish the state of energy, or rather excitement, to which the system had been habituated, and which existed previous to the cessation of the application of diffusible stimuli to the nervous system through the medium of the stomach. The heart and arteries also at length sympathize; the pulse becomes quicker, though it continues small, and the heat of the surface increases. There is, however, throughout the disease, a marked difference between the temperature of the hands and feet and the rest of the body, the former retaining, in some degree, the icy and clammy feel already spoken of, while the rest of the surface may become even hot and dry. If this state continues long without amelioration, a clammy sweat pours from the skin, accompanied with excessive irritability, the disorder of the mind increases, and objects of the most frightful forms present themselves to the imagination of the patient, and in positions in which, as Dr. Pearson says, 'it is physically impossible they can be situated.' I recollect having witnessed a very distressing instance of this sort in the case of a man of the fifth regiment; the unfortunate sufferer, for a considerable time before his death, imagined he saw the devil at the ceiling above his bed; and as the disease, which terminated rapidly, increased, he fancied the evil spirit approached him with a knife to cut his throat; and he actually expired making violent exertions to avoid the fatal instrument."

The mental bias is generally of the melancholic cast, usually concerning some misfortune to which the patient was previously liable. From the moment delirium is fairly established, the patient is deprived of the solace of sleep. Pervigilium may be looked upon as the pathognomonic symptom of the second stage of the disease. "When these symptoms have continued for one, two, or three days, and in a few instances even beyond that period, where a fatal termination is not about to take place, I have almost always observed their gradual mitigation, attended with a strong tendency to sleep, exhibited by yawning and drowsiness, which, as soon as it supervened, became profound, and lasted from six to eighteen hours, and occasionally longer, constituting the *third stage* of this nervous paroxysm, or general relaxation or the nervous energy, similar to the capillary relaxation of the arterial system which takes place during the sweating stage of ague, and to which, in almost every instance that I have seen, convalescence has succeeded."

When the third, or sleeping stage, does not supervene in due course in this disease, and the general symptoms increase in violence, the mind appears to labour under excessive irritation, the patient makes many struggles, which are attended with copious perspirations. These last become cold as the disease advances, the pulse advances in rapidity and becomes thready—the tremor of the hands augments, and extends to the whole frame. Subsultus tendinum, contracted pupils, dry tongue, delirium, &c. usher in the fatal scene, as in typhus fever. Passing over our author's discussions on the proximate cause of delirium tremens, we give the following notes of a dissection, drawn up by Mr. Home, of the 85th regiment.

"On bringing the surface of the brain into view, it did not exhibit any marks of recent inflammatory action; and, with the exception of a small quantity of coagulable lymph, which, on removing the dura mater, was found thrown out between that coat and the tunica arachnoidea, appeared otherwise healthy.

"All the ventricles contained a considerable quantity of serous fluid, but more especially the two lateral, which were very much distended.

"The choroid plexus showed no marks of turgescence. The contents of the thorax and abdomen presented a natural appearance. The liver was small sized, but healthy in its parenchyma."

In respect to the methodus medendi Dr. B. recommends great attention to the stages of the disease, as exhibiting different symptoms, and requiring appropriate remedies. In the first stage, "if slight gastric derangement be present, attended with nausea and occasional vomiting, I have found effervescent draughts, in which there were ten drops of laudanum, administered every second hour, with emollient, and if necessary* anodyne enemata, very efficacious. In the intermediate hour I have been in the habit of giving an oz. of rum with a little warm water and sugar, and of prescribing the warm bath, or tepid affusion, or even the cold affusion, according to the strength of the patient, and the probability of the production of reaction; thus in a young and healthy subject I would have recourse to the cold affusion, and 'vice versa;' but if in this stage the warm bath should be preferred, it ought not to be of a temperature high enough to induce debilitating effects.

"I would recommend that anodyne frictions be at the same time made on the epigastrium, and that the head should be shaved and well rubbed with strong volatile liniment, so as gently to stimulate the surface of the scalp: a blister to the nape of the neck is also of advantage in assisting to excite action in the immediate vicinity of the brain."

When there is no nausea Dr. B. has given an ounce and a half of the camphor mixture, with some æther and ten drops of laudanum instead of the effervescing draught; and, when the appetite permitted it, he allowed soup, arrow-root, or other mild nourishment. The stimulating drink should be of the same kind as that to which the patient had been accustomed previously.

"Should, after all our efforts, the second stage, or that of nervous reaction supervene, we must not be discouraged; but then act on the principles generally recommended, namely, the administration of full doses of opium; taking care, at the same time, to support the efforts of the system by the assistance of diffusible stimuli and antispasmodics, such as brandy, rum, wine, porter, and camphor or musk mixture, with ether, as directed in the first stage, varying, however, their administration according to circumstances. To these means I have been in the habit of adding calomel and Dover's powder, say two grains of the former, and six of the latter, every two hours, until the system became affected, or the disease yielded. Mercury may have been of service here indirectly, owing to its deobstruent and equalizing effects, as well on the circulation in general, as on the secretions.

"The warm bath should also be prescribed with the same views, but particularly to soothe nervous irritation, and favour an equal distribution of the circulating fluids, by exciting general perspiration, during the absence of which cold applications ought to be constantly kept to the head, in order to diminish sensorial action."

We shall terminate this article with an extract from a short essay on the disease under consideration, by Dr. Burton Pearson, of Lazonby, in Cumberland, published 30 years ago, and republished in our esteemed contemporary, the London Medical and Surgical Journal, for May, 1830.

"*Observations on Brain Fever.*

"Brain Fever. At the earnest solicitation of several people who have been afflicted with this grievous malady, I offer this small treatise to the public. Multifarious and repugnant theories on the science of life, still continue to agitate the medical world. Galen's writings, after the extinction of literature for several ages, were again revived, and produced innumerable controversies:—The acrimonious opposition of the Galenists and Arabians, and the complete overthrow of the latter, are well known to every reader. The chemists attacked the Galenists with fury, and they sustained a defeat. Different sects have originated from Stahl, Hoffman, Boerhaave, Cullen, and Brown. A medical review will convince any one how the faculty worry each other at the present day, about their different dogmas, with much injury to themselves and patients. For the above reasons I

* "Reference to my paper, published in the Medical and Surgical Journal for October 1823, will show that I have not copied this practice, I mean the administration of anodyne enemata, from Baron Dupuytren's publication, which appeared some years later than mine."

disavow all theory, and briefly state the circumstances as they occurred to me at the patient's bed-side. Out of 93 cases that have been treated by the principles here adopted, not one has fallen a victim to the disorder; but, when a contrary mode has been attempted, few have recovered, and those only whose constitutions were sufficiently vigorous to resist its ravages.

"I have called it Brain Fever, because it is universally known in Newcastle and its vicinity by that term. The same observation extends to putrid fever.

"*Cause.* — Frequent and excessive intoxication.

"*Description.* — It is preceded by tremors of the hands; restlessness; irregularity of thought; deficiency of memory; anxiety to be in company; dreadful nocturnal dreams, when the quantity of liquor through the day has been insufficient; much diminution of appetite, especially an aversion to animal food; violent vomiting in the morning; and excessive perspiration from trivial causes. The above symptoms increase; the pulse becomes small and rapid, the skin hot and dry; but soon a clammy sweat bedews the whole surface of the body; confusion of thought arises to such a height, that objects are seen of the most hideous forms, and in positions that it is physically impossible they can be situated; the patient generally sees flies or other insects, or pieces of money, which he anxiously desires to possess; and often occupies much time in conversations of negotiation, if he be a commercial man. Often, for many days and nights, he will continue without rest, notwithstanding every effort is made on the part of the physician to appease his mind, by variety of conversation, and variety of stimuli. He frequently jumps suddenly out of bed in pursuit of a phantom, and holds the most ineffable contempt for the practitioner, if he do not concur in his proceedings. He commonly retains the most pertinacious opinion that he is not in his own house, and that some of his dearest relations have sustained a serious injury. During the concourse of these symptoms he often can answer medical questions properly for a short space of time, and then relapses into the raving state.

"*Distinction.* — It is distinguished from putrid fever, in never being contagious, or having purple spots; or ever having a cadaverous smell; or ever being received from human effluvia; and in the delirium being much more impetuous. It is distinguished from inflammation of the brain and its membranes, by not having so vehement a fever, or redness and turgescence of the eye and face; or impatience of light or noise; or hard pulse; and by opposite causes, and opposite method of cure.

"*Method of Cure.*—A full dose of opium should be immediately administered in a glass of wine, and repeated in smaller doses for several hours successively; the quantity of which should be regulated by the constitution of the patient, habit of intoxication, degree of the disease, and other concomitant circumstances. The patient, if he eagerly request it, may be allowed to walk from room to room, and the most consoling language should be used by his attendants;—the debility of the system should be resisted by sherry gruel, cold wine, and porter and soup; which may be given in sufficient quantity, as the stomach in this complaint is very tenacious of what it receives. I never saw the bark, blistering, or affusion of cold water on the head, of any use. Every unnecessary restraint should be carefully avoided; therefore the strait-jacket, which is so universally employed, is the most injurious remedy that can be applied; for, by the perpetual efforts the patient uses to rid himself from confinement, he excites profuse sweating, debilitates the muscular fibre, and soon exhausts the vital principle. If the above mode of treatment be successful, or at least affords the unhappy sufferer a chance or recovery, the impoverishing the system by bleeding, strait-jacket, or abstinence, from invigorating remedies and diet, must be extremely mischievous."

The chief merit of Dr. Blake's essay consists in the division of delirium tremens, (for we will not quarrel about the name) into distinct stages. Indeed if we narrowly watch all diseases, they will be found to consist of many stages of progression, each distinguished by peculiar features, and each demanding some modification of treatment. We have now exhibited the prominent features of the little volume before us, and return thanks to Dr. Blake for the practical information which it contains.

